# ACL reconstruction demonstrates better stability compared to ACL repair for patients with Schenck III and IV knee dislocations

**DOI:** 10.1007/s00402-024-05532-x

**Published:** 2024-09-09

**Authors:** Hendrik Fahlbusch, Peter Behrendt, Jannik Frings, Markus T. Berninger, Alexander Korthaus, Ralph Akoto, Karl-Heinz Frosch, Matthias Krause

**Affiliations:** 1 Department of Trauma and Orthopaedic Surgery, University Medical Center Hamburg-Eppendorf, Hamburg, Germany; 2 Department of Trauma Surgery, Orthopaedics and Sportsorthopaedics, Asklepios St. Georg, Hamburg, Germany; 3 Department of Trauma Surgery, Orthopaedics and Sports Traumatology, BG Hospital Hamburg, Hamburg, Germany; 4 Department of Orthopaedic Surgery, Trauma Surgery, and Sports Medicine, Cologne Merheim Medical Center, Witten/Herdecke University, Ostmerheimer Str. 200, 51109 Cologne, Germany

**Keywords:** Knee dislocation, Internal bracing, Knee dislocation, Multiligament injury, Internal bracing, ACL reconstruction

## Abstract

**Purpose:**

This study compared mid-term outcomes of two anterior cruciate ligament (ACL) restoration techniques within an early total surgical care of acute knee dislocation: repair with additional internal bracing (ACLIB) and reconstruction with autograft (ACLR). Initial results at 12 months demonstrated that ACLR offered superior stability compared to ACLIB.

**Methods:**

Retrospective clinical study of patients with acute type III or IV KD. ACLIB or ACLR procedures were performed accompanied by simultaneous suture and internal bracing of the posterior cruciate ligament (PCL) and repair with lateral augmentation of the medial and lateral complex injuries utilizing Arciero’s reconstruction technique. Patient-reported outcome measurements (PROMs), instrumental stability assessment via the Rolimeter-Test, and stress radiographs were analyzed.

**Results:**

The study involved 20 patients (5 IIIM, 5 IIIL, and 10 IV injuries) with an average follow-up of 35.2 ± 7.4 months. Notable differences in anterior tibial translation on stress radiography favouring ACLR persisted at 24-month follow-up in side-to-side difference (SSD) (ACLIB 2.8 ± 2.5 mm vs. ACLR 0.3 ± 2.6 mm; *p* = 0.0487), but Rolimeter test variance diminished (SSD ACLIB 2.5 ± 0.9 mm vs. ACLR 1.8 ± 1.7 mm). Both groups showed excellent PROMs (Lysholm Score: ACLIB 84.4 ± 15.8 vs. ACLR 89.9 ± 9.0; IKDC Score: ACLIB 77.1 ± 16.2 vs. ACLR 77.7 ± 8.6).

**Conclusion:**

Our results indicate improved anterior stability at 12 months, which persisted at 24 months after ACL reconstruction compared with ACL repair by stress radiography. Both groups showed favourable patient-reported outcomes throughout the follow-up period. Notable rates of postoperative knee stiffness were observed in both groups. These were successfully managed with early, one-time arthroscopic arthrolysis within the first seven months of treatment, resulting in no major range of motion limitations at the 24-month follow-up.

**Level of evidence:**

Retrospective cohort study, III.

**Supplementary Information:**

The online version contains supplementary material available at 10.1007/s00402-024-05532-x.

## Introduction

Knee dislocations (KD) account for only 0.02% of all musculoskeletal injuries but cause significant damage to the knee [5, 20, 31, 35]. Non-surgical approaches have shown unsatisfactory outcomes, leading to a reduced quality of life due to pain and instability [22, 24, 26]. Conversely, surgical intervention is a challenging process, and several surgical techniques have been proposed [10, 22]. These techniques vary from early to late surgery, and involve repair or reconstruction. The surgical procedure can be performed in a single-stage or two-stage process [4]. However, due to the diverse nature of injury patterns, limited case numbers, and the presence of associated injuries, there is a lack of evidence-based treatment recommendations, resulting in controversial discussions regarding treatment options.

In a meta-analysis by Frosch et al. [10] it was reported that acute suture repair yielded favourable clinical outcomes comparable to ligament reconstructions. Building upon this, Heitmann et al. proposed and implemented the concept of additional internal bracing in an early total repair technique for acute KD [13]. A multicentre-study demonstrated promising results for this “ligament bracing” surgical technique [14]. However, despite these advancements, stability assessment following ligament bracing still falls short of the values achieved with single reconstruction of the anterior cruciate ligament (ACL). Studies have reported a failure rate of 16% for primary single ACL repair and internal bracing at a two-year follow-up [19], which has recently increased to 28% at a five-year follow-up [11]. Therefore, the success of ACL ligament repair compared to primary ACL reconstruction in acute KD comes into question. Recently a study conducted by Hecker et al. presented the first midterm-results (follow-up 49 ± 16 months) of patients who underwent surgical treatment for KD involving primary repair and suture augmentation of ACL and PCL [12]. They concluded this surgical technique led to satisfactory clinical results, despite persistent radiological instability and a notable increase in osteoarthritis.

To further explore the comparative efficacy, it is essential to evaluate the longer-term outcome. Our previous research indicated that ACL reconstruction results in superior anterior-posterior stability compared to ACL repair after 12 months [8]. Thus, it becomes crucial to examine the results after 24 months to assess the durability and sustainability of the achieved stability. This comprehensive evaluation is vital in determining the most effective treatment strategy for acute KD.

The main objective of this study was to assess the efficacy of ACL repair with additional internal bracing (ACLIB) in comparison to ACL autograft reconstruction (ACLR) specifically in cases of acute KD.

Our hypothesis posits that despite the potential complications and morbidity associated with autograft harvesting, ACLR will demonstrate superior restoration of anterior tibial translation and yield better overall clinical outcomes.

## Materials and methods

The study received approval from the local ethics committee, and written informed consent was obtained from each patient (2020-10227-BO-ff). All patients were informed about the treatment options and agreed to the specified procedure prior to the operation. Examinations were conducted at 12 and minimum 24 months after index surgery. The results of the 12-month data have been previously published [8].

A total of 23 patients with acute knee injuries were retrospectively included in the study between 2018 and 2021. The treatment of multiligament knee injuries was modified over time based on the clinical experience. Between 2018 and 2020, ACL repair with additional bracing was the chosen approach, while from 2020 onwards, the standard of care shifted to ACL reconstruction using hamstring tendon autografts.

KD cases were categorized according to Schenck’s classification [32]. Only patients with clinical and radiological evidence of acute type III or IV KD were considered for inclusion. Exclusion criteria comprised patients under 18 years of age, polytraumatized individuals, popliteal artery injuries, chronic injuries (older than 12 days), peroneal nerve injuries, and ultra-low velocity injuries in obese patients (grade II according to WHO definition, BMI > 35 kg/m2).

Surgical management was guided by plain radiographs, MRI scans, physical examination of ligamentous instability, and intraoperative findings. ACLIB and ACLR surgical technique was previously described [3, 8, 13, 14], involving arthroscopy for addressing meniscal tears and chondral lesions, followed by open restoration of torn ligaments. In cases of acute type IIIM KD, an anteromedial parapatellar arthrotomy was performed, and for type IIIL or KD IV, an additional lateral incision was made to address the posterolateral corner. Transosseous fixation was employed for arming the cruciate ligament stumps using type 2 FiberWire#2^®^ (Arthrex Inc.). PCL reattachment and internal brace augmentation using FiberTape^®^ (Arthrex) were performed before reattaching the ACL with internal brace augmentation. ACLR cases utilized arthroscopic placement of ACL target wires for subsequent drill channels, and a single-bundle hamstring and anteromedial portal drilling technique was employed.

Subsequent to the restoration of the cruciate ligaments, the management of peripheral ligaments adhered to the principle of “repair what is torn.” Intraligamentous injuries were sutured whenever feasible. For cases involving posteromedial injuries with avulsion of the superficial medial collateral ligament (sMCL), a surgical approach utilizing femoral suture anchors in conjunction with a capsule duplication technique, as described by Hughston et al. [17], was implemented. Posterolateral corner injuries accompanied by popliteus tendon injury were repaired and augmented based on Arciero’s technique [2], employing a hamstring tendon autograft.

Postoperative rehabilitation involved peripheral nerve block anesthesia, followed by standardized physical therapy after 48 h, with limited range of motion and weight bearing for 6 weeks (ex./flex. 0°/0°/90° and 20 kg). Stabilizing braces were worn for 12 weeks, with restricted range of motion for the first 6 weeks.

Building up on our 12-month clinical study clinical testing at 24 months after surgery included functional outcome scoring systems (Lysholm, Tegner, and International Knee Documentation Committee) and pain assessment using the visual analogous scale (VAS). Instrumental measurement using the Rolimeter-Test (Aircast^®^, Neubeuern, Germany) assessed anterior tibial translation. Stress-radiographs (Telos^®^) were performed on both knees to quantify side-to-side differences (SSD). Range of motion (ROM), Dial test, varus and valgus stability and anterior tibial translation were measured using clinical tests such as the Lachman and pivot-shift tests.

Statistical analysis involved presenting data as means and standard deviations (SD). Differences between the groups were assessed using Student’s t-test and Mann-Whitney U-Test for non-parametric parameters. Categorical parameters were compared using Fisher’s exact test. A paired Student’s t-test was performed to assess the differences within the groups after the 12- and 24-month follow-up periods. Statistical analysis was performed using GraphPad Prism 8 (San Diego, CA, US), with significance set at *p* < 0.05. The sample size was determined to be *n* = 20 patients, considering an alpha-error of 5% and test power of 0.8 to detect a 1.5 mm change in the instrumented Lachman test using G-Power (version 3.1.9.7., Heinrich Heine Universität, Düsseldorf) [14].

## Results

### Patient demographics

Demographic data of the included cases are displayed in Table 1. After an average follow-up of 35.2 ± 7.4 months twenty patients were ultimately included in the study. One patient (ACLIB) could not be reached via phone or email and was lost during follow up. Two cases of traumatic ACL re-rupture were identified. The first case involved a female patient (ACLR) who experienced severe valgus trauma during a soccer game, while the second case involved a male patient (ACLIB) injured in a skateboarding accident. Both patients were excluded from clinical assessment and the study.

**Table 1:** Demographic data

Characteristics	Total (*n* = 20)	ACLIB (*n* = 10)	ACLR (*n* = 10)	*p*-value
Female Sex^†^	6 (30)	4 (40)	2 (20)	n.s.
Age^§^	34.0 ± 14.2	36.6 ± 12.9	31.4 ± 15.8	n.s.
Left Knee^†^	14 (70)	5 (50)	9 (90)	n.s.
BMI > 30 kg/m^2†^	4 (20)	3 (30)	1 (10)	n.s.
Follow-up, in months^§^	35.2 ± 7.4	38.5 ± 7.3	32.7 ± 6.0	n.s.
High velocity trauma^†^	9 (45)	5 (50)	4 (40)	n.s.
Schenck Classification^†^				n.s.
▪ Schenck IIIM	5 (25)	3 (30)	2 (20)	
▪ Schenck IIIL	5 (25)	2 (20)	3 (30)	
▪ Schenck IV	10 (50)	5 (50)	5 (50)	
Concomitant injuries^†^				n.s.
▪ Meniscal lesions	10 (50)	5 (50)	5 (50)	
▪ Lig. Patellae	2 (10)	1 (10)	1 (10)	
▪ Posterolateral Capsule or Popliteus complex	6 (30)	1 (10)	5 (50)	

*n* = 20; § mean ± SD; † n (in %); SD standard deviation, ACLIB anterior cruciate ligament internal bracing, ACLR anterior cruciate ligament reconstruction

### Patient reported functional outcome

Table 2 presents the functional outcome scores obtained during the follow-up period. The comparison between the two groups revealed no significant differences in terms of the VAS, Tegner Score, Return to Sports, and functional scores. The IKDC and Lysholm Score displayed higher values at the 24-month follow-up compared to the 12-month follow-up.

**Table 2:** Functional outcome scores

Parameters	Total (*n* = 20)	ACLIB‡ (*n* = 10)	ACLR‡ (*n* = 10)	*p*-value‡
VAS rest^§^	0.3 ± 0.7	0.1 ± 0.3	0.4 ± 1.0	n.s.
VAS exercise^§^	1.7 ± 1.7	1.3 ± 1.5	2.0 ± 1.9	n.s.
Lysholm Score^§^ Δ	2.8 ± 4.1	2.4 ± 4.3	3.2 ± 4.2	n.s.
Lysholm Score^§^	88.3 ± 11.1	84.4 ± 15.8	89.9 ± 9.0	n.s.
Subjective IKDC Score^§^ Δ	3.8 ± 8.1	6.7 ± 9.9	0.9 ± 4.7	n.s.
Subjective IKDC Score^§^	77.4 ± 12.6	77.1 ± 16.2	77.7 ± 8.6	n.s.
Tegner Score^§^				
▪ Preoperative	6.4 ± 2	6.2 ± 1.5	6.6 ± 2.4	n.s.
▪ Delta Δ (pre-post)	1.6 ± 1.5	1.3 ± 1.6	1.8 ± 1.4	n.s.

*n* = 20; § mean ± SD; † n (in %); Δ indicates the delta between 12-month and 24-month follow-up; SD standard deviation, ACLIB anterior cruciate ligament internal bracing, ACLR anterior cruciate ligament reconstruction, VAS visual analogous scale, IKDC International Knee Documentation Committee (IKDC)

### Clinical testing and instrumental stability testing

Table 3 presents the data obtained from clinical examination and instrumental testing during the follow-up period. At the 24-month follow-up, the difference persisted in stress radiography (24-month SSD stress radiography ACL: ACLIB 2.8 ± 2.5 mm vs. ACLR 0.3 ± 2.6 mm; *p* = 0.0487), while the difference in anterior tibial translation in Rolimeter examination decreased, mainly due to a slight increase in SSD in the ACLR group compared to the 12-month follow-up (24-month Rolimeter SSD: ACLIB 2.7 ± 1.5 mm vs. ACLR 1.3 ± 1.3 mm; *p* = 0.0339) [8]. ACLIB group showed a slightly greater valgus gapping (*p* = 0.0147). Furthermore, the maximum flexion angle of both groups increased during the follow-up period (12-month Flexion 125.5 ± 11.8° vs. 24-month Flexion 128.4 ± 10.2°; *p* = 0.0023).

**Table 3:** Clinical examination and instrumental stability assessment at the follow-up

Parameters	Total (*n* = 20)	ACLIB (*n* = 10)	ACLR (*n* = 10)	*p*-value‡
Flexion^§^ Δ in °	4.1 ± 5.2	3.0 ± 3.5	5.2 ± 6.5	n.s.
Flexion^§^, in °	128.4 ± 10.2	132.0 ± 10.1	124.7 ± 9.5	n.s.
Flexion SSD^§^, in °	10.3 ± 9.2	8.5 ± 10.8	12.1 ± 7.4	n.s.
Extension deficit^§^, in °	-0.3 ± 2	0	-0.5 ± 2.8	n.s.
Dial test (IR) SSD^§^ in °	0.2 ± 4.9	-0.1 ± 5.2	0.5 ± 4.7	n.s.
Dial test (ER) SSD^§^ in °	-0.5 ± 2.8	-0.8 ± 3.4	-0.2 ± 2.5	n.s.
Medial instability^†^				
▪ 2–5 mm	7	4	3	n.s.
▪ 5–10 mm	1	1	0	n.s.
▪ > 10 mm	0	0	0	
Lateral instability^†^				
▪ 2–5 mm	7	5	2	**0.0147**
▪ 5–10 mm	0	0	0	
▪ > 10 mm	0	0	0	
Instrumental testing (Rolimeter)^§^				
▪ ACL SSD Δ in mm	0.1 ± 1.1	-0.2 ± 1.0	0.5 ± 1.1	n.s.
▪ ACL SSD in mm	2.1 ± 1.4	2.5 ± 0.9	1.8 ± 1.7	n.s.
Stress Radiography (Telos^®^)^§^				
▪ ACL SSD Δ in mm	-0.1 ± 0.4	0.1 ± 0.2	-0.3 ± 0.4	n.s.
▪ ACL SSD in mm	1.5 ± 3.0	2.8 ± 2.5	0.3 ± 2.6	**0.0487**
▪ PCL SSD Δ in mm	-0.1 ± 0.4	0.1 ± 0.4	-0.3 ± 0.5	n.s.
▪ PCL SSD in mm	4.1 ± 2.1	4.9 ± 2.3	3.4 ± 1.7	n.s.
Lachman test^†^				n.s.
▪ Grade I	11 (55)	5 (50)	6 (60)	
▪ Grade II	5 (25)	4 (40)	2 (20)	
Grade of pivot-shift test^†^				n.s.
▪ Absent ▪ Grade 1 (glide)	18 2 (10)	9 (90) 1 (10)	9 (90) 1 (10)	

*n* = 20; § mean ± SD; † n (in %); Δ indicates the delta between 12 month and 24 month follow-up; bold p value indicates statistical significance with *p* < 0.05; SD standard deviation, ACLIB anterior cruciate ligament internal bracing, ACLR anterior cruciate ligament reconstruction, SSD site-to-side difference, IR internal rotation, ER external rotation

There were no significant differences between the two groups in terms of extension, varus thrust, Pivot-Shift, and Dial test (external and internal rotation). At the final follow-up, two patients from the ACLR group reported subjective instability, which could not be confirmed by objective instrumental stability testing.

### Complications

Postoperative impaired range-of-motion, defined as a flexion angle of less than 90° and/or an extension deficit greater than 10°, was observed in seven cases (four in the ACLIB group and three in the ACLR group). Among these cases, four were categorized as type IV injuries, two as type IIIL, and one as type IIIM injuries. Despite undergoing aggressive physiotherapy, all patients failed to show improvement, necessitating the implementation of early arthroscopic lysis of adhesions and debridement (LOA). The average time to LOA intervention was 17.4 ± 8.0 weeks, and following LOA, all patients demonstrated a ROM greater than 0/0/120° at the 12-months follow-up with no need for another intervention until their final follow-up assessment. In addition to the two instances of traumatic ACL rerupture, a patient in the ACLR group encountered a traumatic meniscus rupture, which was subsequently surgically repaired. Upon examination, all ligaments were found to be structurally intact, rendering this patient eligible for inclusion in the study.

## Discussion

Main results indicate an enhanced anterior stability after 12 months, which prevailed in the 24-months follow-up following ACL reconstruction compared to ACL repair according to stress radiography. However, instrumental testing (Rolimeter) suggested that the ACL graft became softer compared to the 12-months follow-up. Notably, both groups displayed enhanced maximum flexion and patient-reported outcome scores over the follow-up period, collectively contributing to commendable mid-term clinical outcomes. Additionally, a considerable rate of re-operation mainly due to postoperative knee stiffness was noted in both groups in the 12-months follow-up. However it is noteworthy, there were no further surgical interventions after the first follow-up examination. Revision surgery was necessary in 40% of cases, all of which occurred within the first 7 months after the index surgery.

The management of torn ACL in the context of multiligament knee injuries has sparked a prolonged debate [10, 22, 37]. Few studies have explored ACL reconstruction in acute KD within an early total repair approach [9, 12, 16, 26]. Notably, no precise comparative analysis between ACL reconstruction and repair has been conducted. Our previous research indicated that ACL reconstruction results in superior anterior-posterior stability compared to ACL repair at 12 months [8]. At the 24-month follow-up, objective stress radiography demonstrated superior anterior-posterior stability in the ACLR group compared to a reduction in instrumental anterior-posterior stability similar to the ACLIB group. This discrepancy may be due to the gradual loosening of the ACL autograft, which reduces the initial stability. Key factors include the healing of the tendon graft within the bone tunnels and the remodelling of the graft’s substance, often referred to as ‘ligamentization‘ [30, 34, 39].

Changes in instrumented laxity measurements have been described in a follow-up study (1, 2 and 5 years) comparing dynamic ACLIB and ACLR, which was not associated with clinical scores [11]. In similarity to our study, SSD was < 3 mm in both groups, indicating no anterior knee instability in both groups [1, 21, 23].

Clinically, there was minimal difference and satisfactory anterior-posterior stability in both groups. Nonetheless, the ACLR graft appears to exhibit primarily greater stability, potentially reducing the re-rupture rate, a risk heightened in this period as seen in studies of isolated ACL rupture [29]. Conversely, besides possible donor side morbidity by harvesting the autograft, repair might offer improved proprioceptivity due to partial preservation of cruciate ligament remnants and thus leading to superior clinical results. However, this study did not present evidence supporting this claim.

It’s important to recognize that stress radiographs were taken at 90° flexion, while Rolimeter measurements were conducted at 20–30° flexion. Biomechanical studies indicate that at 90° flexion, anterior-posterior stability additionally relies on peripheral structures such as the anterior lateral ligament (ALL) and sMCL [15, 38].

Aligned with our findings, Hirschmann et al. [16] indicated a positive association between ACL reconstruction and clinical outcomes. Variations might stem from different bracing techniques and ACL injury patterns; proximal tears could outperform midsubstance or distal tears [33, 36]. A study investigating isolated ACL injuries, revealed an anterior-posterior translation of 1.9 mm SSD for ACL repair and 0.9 mm SSD for ACL reconstruction [19]. However, there was no significant difference in subjective outcome scores between the two groups. Internal bracing using rigid suture augmentation led to a 3.3 mm anterior-posterior SSD for ACL, along with IKDC and Lysholm scores above 80 [14], echoing Rosteius et al. [27]. Hecker et al. [12], in a study with 49-month follow-up treated KD with primary repair and suture augmentation of ACL and PCL, demonstrated a 2 mm anterior-posterior translation SSD using KT-1000, stress radiography SSDs of 4 mm for ACL and 7 mm for PCL, along with Lysholm Score of 84 and IKDC of 73 points. These findings closely resemble the outcomes of our ACL repair procedures, although they slightly lag behind the stability and PROMs of ACL reconstruction.

Few studies reported Lysholm scores > 89, as observed in our ACL reconstruction group [10, 12, 14, 16, 27]. Traumatic graft failure occurred in 1/11 ACL grafts in both groups following proper return-to-play rehabilitation. Graft failure and clinical outcomes correlate with patient age and activity level [18, 25, 28]; our active, mid-age population showed similar tendencies. Recent ACL reconstruction and repair studies reveal higher graft failure in young, active patients undertaking high-risk pivoting sports suggesting an ACL treatment selection dependent on patient demand [19, 28].

However, substantial stiffness and subsequent LOA rates are acknowledged in both groups. Compared to a systematic review of our group [7], this study had higher LOA rates due to liberal early LOA indications, supported by improved range of motion and functional scores [6]. Stiffness was addressed early in rehabilitation, with LOA after an average of 17.4 weeks. Intriguingly, maximum flexion angle increased over follow-up, emphasizing early intervention for significant stiffness, while minor ROM restrictions can be addressed successfully through rehabilitation even after 12 months.

Limitations: Conclusions based on this study are limited by the relatively small case number and inhomogenous injury patterns. Improved comparability was tried to achieve by exclusion of obese patients, accompanying fractures of the tibial plateau, major nerve and vascular injuries. Decision for ACL treatment was not randomized, but changed during the study time with ACL reconstructions performed in the second half of the study period. In addition, no matched-pair analysis was feasible given the great rarity of acute KD. Long-term follow up is necessary to validate the concept of primary ACL reconstruction as recent studies have shown the increase of graft failure during the observation time.

## Conclusion

Our results indicate improved anterior stability at 12 months, which persisted at 24-months after ACL reconstruction compared with ACL repair by stress radiography.

Both groups showed favourable patient-reported outcomes throughout the follow-up period, with a trend towards greater improvement in the ACLR group. Significant rates of postoperative knee stiffness were noted in both groups, which were successfully addressed with early and one time LOA within the first 7 months, resulting in no significant ROM limitations at follow-up.

## Electronic supplementary material

Below is the link to the electronic supplementary material.

Supplementary Material 1

Supplementary Material 2

Supplementary Material 3

Supplementary Material 4

Supplementary Material 5

Supplementary Material 6

Supplementary Material 7

Supplementary Material 8

## Data Availability

The data that support the findings of this study are available upon reasonable request from the correspondingauthor [MK]. The data are not publicly available due to containing information that could compromise the privacy ofresearch participants.

## Abbreviations

ACL: Anterior cruciate ligament.
ACLIB: Anterior cruciate ligament internal bracing.
ACLR: Anterior cruciate ligament reconstruction.
BMI: Body mass index.
IKDC: International Knee Documentation Committee.
KD: Knee dislocation.
LOA: Arthroscopic lysis of adhesions.
PCL: Posterior cruciate ligament.
ROM: Range of motion.
SD: Standard deviation.
sMCL: Superficial medial collateral ligament.
SSD: Side to side difference.
VAS: Visual analog scale.
